# Using proton pump inhibitors increases the risk of hepato-biliary-pancreatic cancer. A systematic review and meta-analysis

**DOI:** 10.3389/fphar.2022.979215

**Published:** 2022-09-14

**Authors:** Wence Zhou, Xinlong Chen, Qigang Fan, Haichuan Yu, Wenkai Jiang

**Affiliations:** ^1^ First Clinical Medical College, Lanzhou University, Lanzhou, Gansu, China; ^2^ Department of General Surgery, Second Hospital of Lanzhou University, Lanzhou, Gansu, China; ^3^ Department of General Surgery, First Hospital of Lanzhou University, Lanzhou, Gansu, China

**Keywords:** drug side effects, observational studies, proton pump inhibitor (PPI) therapy, malignant tumor, dose—response

## Abstract

**Background:** More and more studies are focusing on the adverse effects and damage caused by PPI abuse, we carried out a systematic review and meta-analysis for assessing whether the proton pump inhibitor (PPI) leads to hepato-biliary-pancreatic cancer.

**Methods:** PubMed, EMBASE and Web of Science were searched until 1 July 2022, 25 studies (17 case-control and 8 cohort studies; 2741853 individuals) included in this study. Pooled Odd Ratios (ORs) were used for random effect models. Sensitivity analysis and dose-response analysis, subgroup analysis were all conducted.

**Results:** The aggregate OR of the meta-analysis was 1.69 (95% confidence interval (CI): 1.42–2.01, *p* = 0.01) and heterogeneity (*I* ^*2*^ = 98.9%, *p* < 0.001) was substantial. According to stratified subgroup analyses, the incidence of hepato-biliary-pancreatic cancer was associated, expect for study design, study quality and region. Risk of hepato-biliary-pancreatic cancer is highest when people is treated with normal doses of PPI. The risks decrease and become insignificant when the cumulative defined daily dose (cDDD) increases.

**Conclusion:** The use of PPI may be associated with an increased risk of hepato-biliary-pancreatic cancer. Hence, caution is needed when using PPIs among patients with a high risk of hepato-biliary-pancreatic cancer.

## Introduction

Proton pump inhibitors have been the most widely used drugs since the 1980s for treating gastroesophageal reflux disease (GRED), H. pylori infection, Zollinger-Ellison syndrome and a number of other diseases caused by hyperacidity irreversibly inhibiting H+/K+-ATPase in stomach cell (Targownik et al., 1528). These diseases usually require long-term treatment, which leads to the consequence of overdose and adverse effects. Current research suggests that changes in the gut microbiome and dysbiosis are closely related to the development of malignant tumors (Xia et al., 1471; Zhu et al., 1476; Huang et al., 1557), and that PPI can alter the pH of the digestive tract, affecting normal cell metabolism and flora migration, causing abnormal metabolism in adjacent organs and producing carcinogenic inflammatory factors, leading to tumor development (Jang et al., 1536; Imhann et al., 1468).

Hepato-biliary-pancreatic cancers, as the most important malignant tumors of the abdomen, are characterized by the influence of microbiome of hepatopancreatic ampulla and biliary tract and are significantly more difficult to treat and have a poorer prognosis than other malignant tumors (Mao et al., 2021; Wheatley et al., 1532; Nadeem et al., 2021). Although some studies have suggested that PPIs can induce tumors in the liver, gallbladder and pancreas by affecting duodenal and biliary tract microbiome (Blackler et al., 2015; Yang et al., 2020), the current clinical studies are not sufficient to support this opinion. Hence, and high-quality evidence of an association between the use of PPI and hepato-biliary-pancreatic cancer is needed. Therefore, evidence is needed to help doctors address the adequacy of the prescription and the patient’s dose abnormalities during treatment.

We have clarified the association between PPI and risk of hepato-biliary-pancreatic cancer by including known studies in this meta-analysis. We likewise evaluated whether the risk of hepato-biliary-pancreatic cancer aggravates when the dose of PPI increases.

## Methods

### Search strategy

This study is based on the aloe system assessment and analysis criteria (PRISMA) and the optional reporting items in the Corcoran manual (Supplementary Appendix S1). This research plan registered in the international prospective systematic evaluation register is NO. CRD 420211103, and can access the PubMed, EMBASE and web of science databases, research collections of proton pump inhibitors and hepato-biliary-pancreatic cancer, as well as comparative studies, this was done by the two researchers themselves. The search strategy is set out (Supplementary Appendix S2). In order to identify other articles, additional manual searches were conducted for references in research reports and related reviews

### Inclusion and exclusion criteria

Including criteria: (Targownik et al., 1528) Observational studies with a history of PPI drug use as an intervention and pancreatic cancer, bile tract cancer, and hepatocellular as outcome indicators, including case-control (pooled analysis of nested case-control and case-control studies) or cohort studies (pooled analysis of cohort studies); (Xia et al., 1471) Exact records of PPI users; (Zhu et al., 1476) Defined results of pancreatic, liver and biliary carcinoma; (Huang et al., 1557) Odds ratio (OR), Relative risk (RR) and hazard ratio (HR) reported for selected neoplasms and 95% CI.

Excluding criteria: (Targownik et al., 1528) literature review or comments; (Xia et al., 1471) evaluate cancer recurrence or survival. We did not exclude based on the quality of the literature; therefore, no studies were excluded due to poor study design or low data quality.

### Data extraction

Two auditors independently examined titles meeting the including and excluding criteria and examined whether the information of study is insufficient. Subsequently, the full text of the selected articles was evaluated and two auditors extracted critical information which includes first author, year of publication, region/country, study design, exposure definition, cDDD independently. Any disagreement has been resolved by consensus between the two auditors or arbitrated by the third auditor. The quality of the observational studies was assessed by two authors using the Newcastle-Ottawa scale.

### Data analysis and integration

ORs were used as a common measure of association between studies. Statistical analyses were performed using R (4.2.1). We derived aggregated risk estimates, which were expressed with 95% CI in total hepato-biliary-pancreatic cancer and in each cancer. Random effect models were used to take into account the heterogeneity of aggregate estimates. We used Cochran Q test to evaluate heterogeneity between studies, quantified using Cochran *Q* and *I* ^*2*^ statistics.

Subgroup analysis and meta-regression was performed (classification by study design [case-control or cohort), region (Western or Asia), and the Newcastle-Ottawa Scale (NOS) score (<7 or ≥7)].

We also investigated a potential nonlinear dose-response relationship between cDDD and hepato-biliary-pancreatic cancer via restricted cubic splines and fractional polynomial models reported in Bagnardi et al. (1093). Dose-response study was conducted by mean cDDD values reported in the included articles.

## Results

### Description of included studies

**FIGURE 1:**
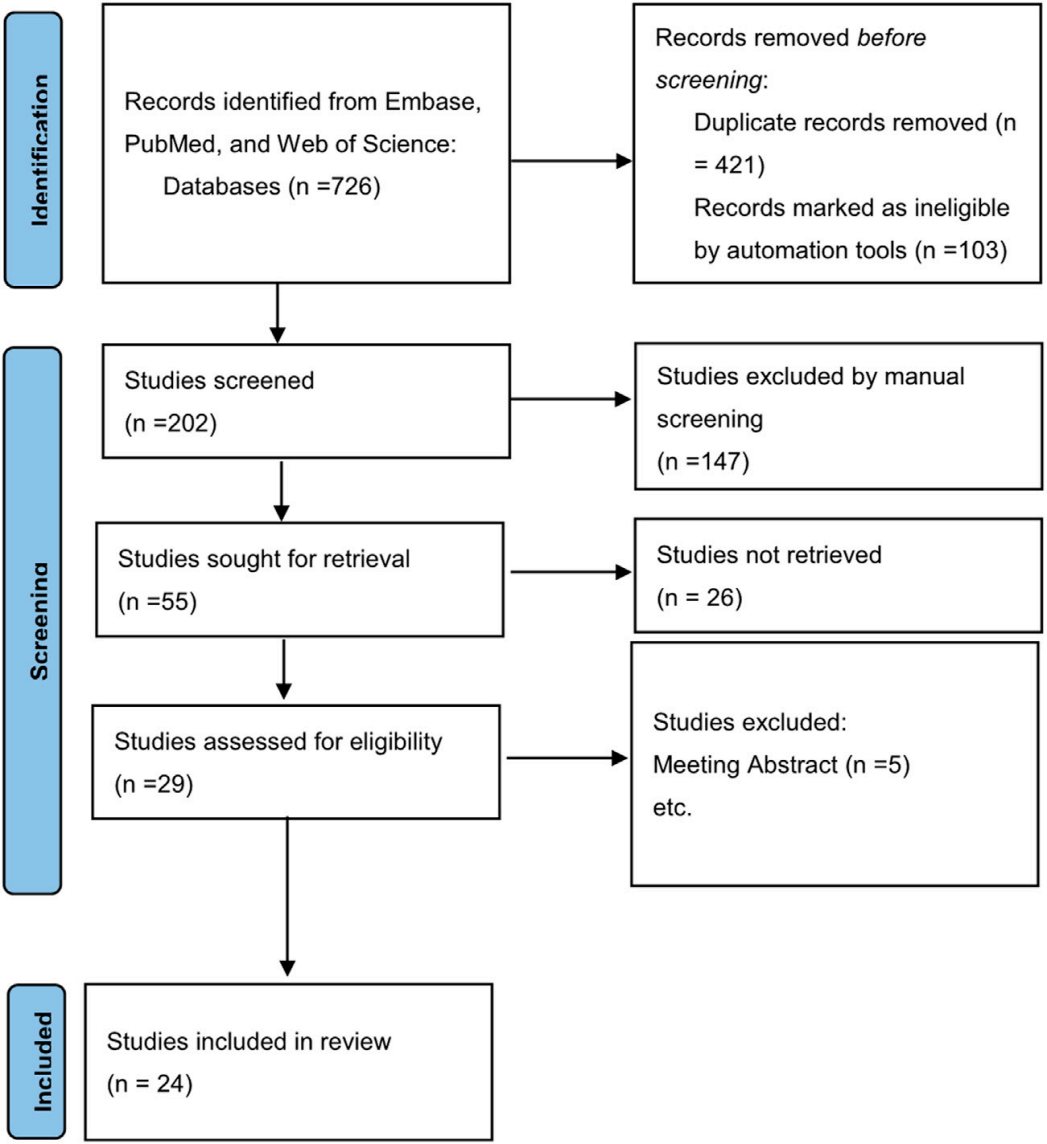
A flow diagram demonstrating the search strategy and study selection process for this study.

The electronic searches resulted in 726 titles, of which 25 studies met the inclusion criteria for the meta analysis. 4 article did not meet the inclusion criteria for meta-analysis because they were review articles, the flowchart of study selection was demonstrated in Figure 1. 25 articles and a population of 2741853 individuals were finally included in our analysis, an article of these contains 2 population-based studies (Primary Care Clinical Informatics Unit [PCCIU] and UK biobank studies) which has different study designs, many of the original studies included in this analysis had different subgroup analyses. Therefore, we analyzed them separately in our article. Supplementary Table S1 provides the details of the study characteristics. Supplementary Table S2 demonstrates the quality assessment and New-castle Ottawa scale scores of the included studies. Scored 7 is considered as high-quality in our study.

### Association between proton pump inhibitor use and hepato-biliary-pancreatic cancer

**FIGURE 2:**
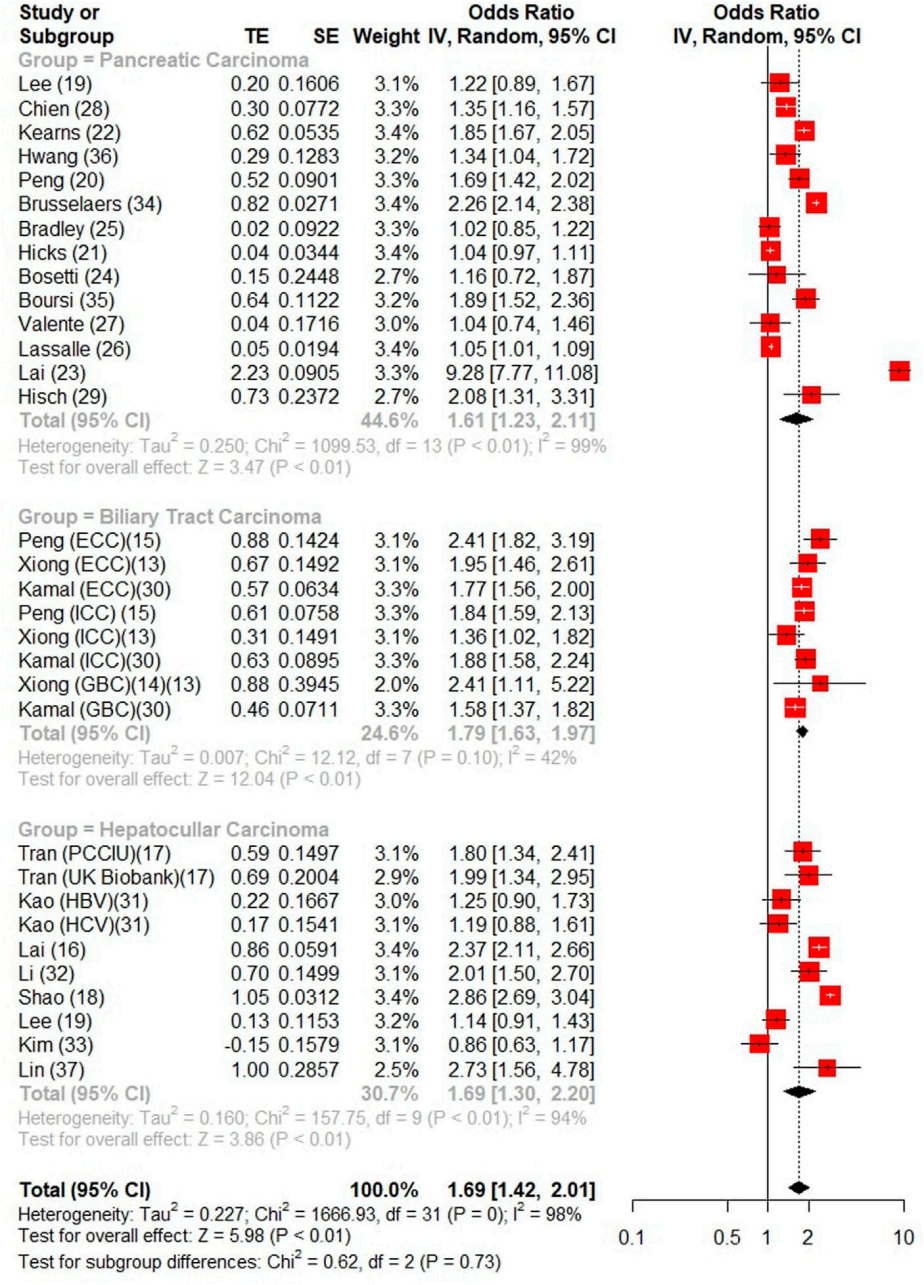
Forest Plot of Studies analyzing relationship between long-term PPI use and Hepato-Biliary-Pancreatic cancer.

All of the 25 studies (17 cases control (Bradley et al., 2012; Bosetti et al., 2013; Lai et al., 2013; Lai et al., 2014; Risch et al., 2015; Chien et al., 2016; Kearns et al., 2017; Valente et al., 2017; Peng et al., 2018a; Peng et al., 2018b; Hicks et al., 2018; Shao et al., 2018; Tran et al., 2018; Xiong et al., 2020a; Xiong et al., 2020b; Lee et al., 2020; Lassalle et al., 2022) and 8 cohort studies (Boursi et al., 2017; Hwang et al., 2018; Li et al., 2018; Kao et al., 2019; Brusselaers et al., 2020; Lin et al., 2020; Kamal et al., 2021; Kim et al., 2022) contained association between PPIs and hepato-biliary-pancreatic cancer risk. Results from both cohort and case-control studies were reported in two studies (Kearns et al., 2017; Tran et al., 2018), four studies report an association between PPI and multiple tumors (Chien et al., 2016; Xiong et al., 2020a; Lee et al., 2020; Kamal et al., 2021). An aggravated risk and the subgroup analysis by the types of pooled estimates indicated that the pooled estimates were similar numerically and can be seen in Figure 2, with significantly increased risks in studies reporting ORs (OR = 1.69, 95%CI:1.42–2.01). *I* ^*2*^ > 50% was found by heterogeneity test, we identified evidences of publication bias by the visual the results of Egger test (*p <* 0.001) (Supplementary Figure S1), corrected (OR = 1.26 95% CI: 1.05–1.52) after adding 13 studies by subtractive complementation using trim and filling method. The heterogeneity was obvious (Cochran’s Q = 4478.36, *I2* = 98.9%, *p* < 0.001). Subgroup analysis and meta-regression in different subgroups were performed (Supplementary Table S3). Differences in study design, region, and NOS score were not sources of heterogeneity. An overall sensitivity analysis revealed that the heterogeneity originated from the study of Brusselaers et al. (2020) and Lassalle et al. (2022) and Shao et al. (2018) (Supplementary Figure S2), a rereading of these studies revealed that the Brusselaers’ study was biased by a smaller sample size, while the latter two were biased by different definitions of long-term PPI use. When these studies were excluded and re-analyzed, it was found that *I* ^*2*^ *<* 50%.

### Proton pump inhibitor use and different cancers

A total of 12 studies reported the risk of liver cancer (3 studies reported intrahepatic bile duct cancer as an outcome and 9 studies reported the risk of hepatocellular carcinoma), which were analyzed separately and found that long-term PPI use increased the risk of liver carcinoma (OR = 1.69, 95% CI: 1.37–2.08), hepatocellular carcinoma (OR = 1.69, 95% CI: 1.30–2.20) were at risk of development. six case-control studies and five cohort studies (one case-control study reported the results of the cohort study) showed a significant association between long-term PPI use and the development of liver cancer when subgroup analysis was performed (OR 1.91, 95% CI:1.48–2.46 for case-control, OR = 1.51, 95% CI: 1.09–2.07 for the cohort study), and the risk of increased incidence of liver cancer after long-term PPI use was found in different geographical regions (Asia: OR = 1.75, 95% CI: 1.39–2.21; Western: OR = 1.66, 95% CI: 1.30–2.13) (Supplementary Table S3).

A total of 4 studies reported the risk of long-term PPI use and the risk of biliary system carcinoma, of which 2 reported the risk of gallbladder cancer (OR = 1.63, 95% CI: 1.31–2.04) and 3 reported the risk of intrahepatic and extrahepatic biliary tract cancer (OR = 1.83, 95% CI: 1.60–2.09), respectively, while the long-term use of PPI was associated with the overall risk (OR = 1.79, 95% CI: 1.63–1.97), and long-term PPI use was significantly associated with the overall biliary tract cancer (OR = 1.79, 95% CI: 1.63–1.97).

15 studies (13 case-control, 2 cohort studies) showed that long-term use of PPI drugs increased the risk of developing pancreatic malignancies (OR = 1.61, 95% CI: 1.23–2.11) and that PPI uses increased the incidence of pancreatic tumors in different geographical populations (Asia: OR = 1.67, 95% CI: 1.29–1.77; Western: OR = 1.45, 95% CI: 1.17–1.78).

### Cumulative defined daily dose, duration of proton pump inhibitor uses, and cancer risks

**FIGURE 3:**
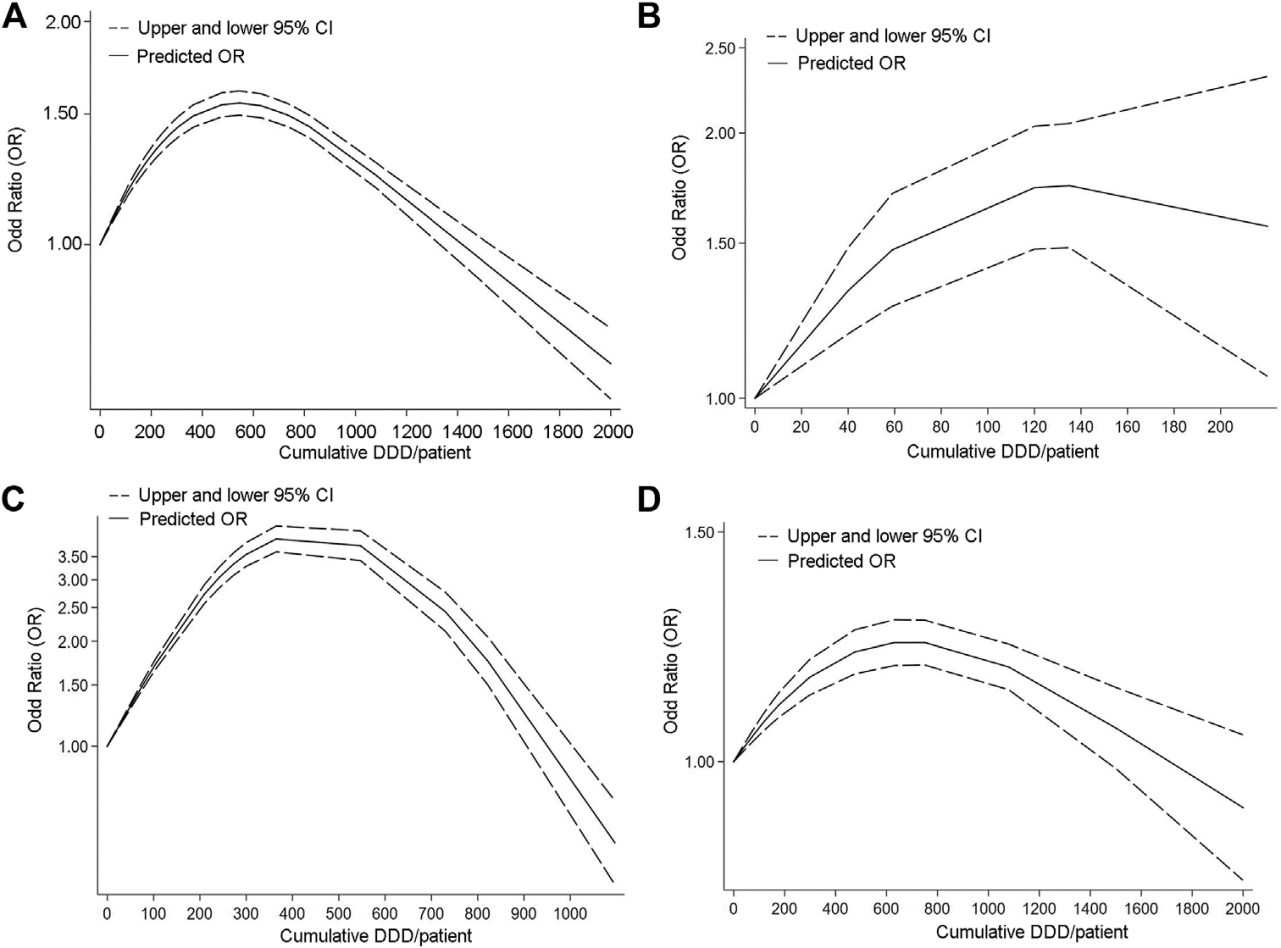
Dose response curve of Studies analyzing relationship between long-term PPI use and Hepato-Biliary-Pancreatic cancer. [**(A)** Hepato-Biliary-Pancreatic cancer. **(B)** biliary tract cancer. **(C)** hepatocellular carcinoma. **(D)** pancreatic cancer].

The WHO developed the anatomical therapeutic chemical (ATC) classification system in 1969, which established the defined daily dose (DDD) as the unit of medication frequency analysis. It is defined as the average daily dose of a drug used for the primary therapeutic purpose in adults (Lei et al., 2021). Information on hepato-biliary-pancreatic cancer risks correlated with cDDD of PPIs was provided in ten studies (Figure 3).

In order to better assess the association between cDDD and hepato-biliary-pancreatic cancers, we analyzed the association between cDDD and overall tumor risk in a comprehensive manner. The association of dose change with the risk of pancreatic cancer, hepatocellular carcinoma and biliary tract cancer was then analyzed separately.

Fourteen studies provided information on hepato-biliary-pancreatic cancer risks correlated with cDDD of PPIs.

The OR was highest at about 500 cDDD/per patient (Figure 3A), which may mean that long-term normal dose of PPI use makes the risk of malignant tumor development significantly higher. The OR declined and became not apperant at around 1400 cDDD per patient or higher. The risk of hepatocellular and pancreatic cancers is highest at a cDDD of 300–400, while the risk of bile duct cancer is highest at a cDDD of about 120. The results of the dose-response study for hepatocellular carcinoma and pancreatic cancer were similar to the overall results.

## Discussions

Current guidelines for GERD, hemorrhagic ulcers and H. pylori infection use IPPs as the drug of choice, but PPI also have the potential to alter the structure of upper gastrointestinal pH, which has potential links to previous studies on the pathogenesis of hepato-biliary-pancreatic cancer, and our study supports the view that excessive use of PPI increases the risk of hepato-biliary-pancreatic cancer. This may lead clinicians to be more careful in choosing indications and to control the dose of the drug so that the disease population is treated properly without worrying about increased tumor risk.

After analyzed 25 studies using a random effects model, including a total of 2741853 patients. This meta-analysis aimed to clarify that long-term use of PPI may increase the risk of hepato-biliary-pancreatic cancer.

Based on the results of our study, a normal dose of PPI is associated with an increased risk of developing hepato-biliary-pancreatic cancer. However, as the dose increases, the positive correlation between the two diminishes (cDDD > 2000/per patient).

Several mechanisms suggest a potential oncogenic effect of PPI in hepato-biliary-pancreatic cancer. These effects include an increase in levels of abnormal gastrointestinal hormones and intestinal microbiota, as well as the production of carcinogens.

### Abnormal levels of gastrin and cholecystokinin

Prolonged use of PPI leads to a rise in gastric pH and an increase in gastrin production by G-cells with negative feedback. In addition to stimulating the secretion of digestive glands and accelerating nutrient absorption, gastrin seems to induce the development and growth of gastrointestinal cancers by binding to CCK-BR on the surface of the enterochromaffin-like cells (ECL) (Smith et al., 1995). In hepatocellular carcinoma, CCK-BR and a precursor form of gastrin are expressed in tumor cells (Caplin et al., 1999), and this expression may be associated with apoptosis (Savage et al., 2006). Gastrin-releasing peptide promotes hepatocellular carcinoma cell growth not only by interacting with homologous receptors of gastrin-releasing peptide co-expressed in tumor cells but also by activating the mitogen-activated protein kinase/extracellular signal-regulated kinase 1/2 (MAPK/ERK1/2) pathway through a non-dependent mechanism of the epidermal growth factor receptor (EGFR) (Savage et al., 2006), it can also inhibit the growth of normal liver cells by blocking the activation of ER (Li et al., 2013). A DNA vaccine targeting gastrin-releasing peptide has been shown to inhibit the growth of blood vessels in liver tumors and to destroy tumor cells (Meko’o et al., 2014; Stubbs et al., 2002). However, in cholangiocarcinoma, gastrin appears to have the opposite effect to that of hepatocellular carcinoma, inhibiting the proliferation of cholangiocarcinoma cells and inducing apoptosis via the Ca2+ dependent protein kinase C (PKC)-α pathway (Kanno et al., 2001). However, when gastrin receptors are the target of pancreatic cancer treatment, specific antagonists can inhibit the growth of pancreatic cancer cells by blocking the cellular stimulatory effect of gastrin. Current clinical studies have demonstrated that these drugs have the potential to prolong survival and are no less effective than conventional treatments for pancreatic cancer (Chau et al., 2006), however, further research is needed on their safety and long-term efficacy (Morisset et al., 2004).

A current study has proved long-term PPI use may pose a risk for gallbladder dysfunction and biliary complications (Cahan et al., 2006), a retrospective analysis of stone recurrence in patients after endoscopic retrograde cholangiopancreatography (ERCP) found that PPI may pose a risk for recurrence of common bile duct stone (CBDS) in ERCP patients (Fukuba et al., 2017). Long-term PPI use may be associated with the abnormal secretion of CCK, a gastrointestinal peptide released from the upper part of the small intestine, which has a similar peptide structure to gastrin. CCK has functions that include stimulation of intestinal motility, stimulation of pancreatic enzyme secretion, and stimulation of gastric acid secretion (Rehfeld, 2017). Its primary function is to trigger gallbladder emptying by binding to the CCK A-type receptor (CCKAR) and mediating the activation of post-membrane signaling pathways in smooth muscle, defects in CCKAR are a key point of impairment of gallbladder motility, which in turn may form the background for GBC (Suzuki et al., 2001; Best Practice & Research Clinical Endocrinology & Metabolism, 2008), abnormal level of CCK results in reduced or delayed postprandial gallbladder contraction, leading to bile stagnation and creating an environment for cholesterol supersaturation and subsequent gallstone formation (Savage et al., 2006; Meko’o et al., 2014; Stubbs et al., 2002; Kanno et al., 2001; Chau et al., 2006). Both CCK and its receptor CCKAR are important in the pathogenesis of biliary tract tumors, CCK is currently thought to exhibit growth-stimulating effects on biliary tract-derived cancer cell line (Lee et al., 1989), an analysis of the biliary tract tumor in Shanghai, China, found that women with the CCKAR genotype were at increased risk of gallbladder cancer, and biliary tumorigenesis may be inhibited when CCKAR is in an antagonistic state (Ogura et al., 2002). However, CCKAR receptors are more highly expressed in patients with cholelithiasis than in the normal population, while CCKAR expression is reduced in patients with GBC (Faridi et al., 2015), it may be because CCK remains chronically high in CA patients, leading to a decrease in receptor number and activity responsiveness. However, Kazmi observed a significant increase in CCKAR mRNA and protein expression in GBC tissues (Kazmi et al., 2014). Furthermore, gastrin or CCK showed a definite growth stimulating effect on biliary tract-derived cancer cell lines, and CCKAR and CCK-BR mRNA were detected in all biliary and pancreatic cancer (Jang et al., 2005). CCK also has a pro-pancreatic function in the normal gastrointestinal environment, and high CCK levels have been found to stimulate abnormal pancreatic growth and promote early carcinogenesis and malignant tumor growth by binding to CCKAR, pro-carcinogenic effect of CCK can be inhibited by antagonizing CCKAR (Smith et al., 1990). Although CCKR expression has been widely reported in many tumors (Caplin et al., 1999), relevant studies have shown that none of the cancer samples had statistically higher CCKR expression than all normal samples (Roy et al., 2016). Therefore, the association between CCK and Hepato-biliary-pancreatic cancer still needs to be further investigated (Srivastava et al., 2008).

### Abnormal gut microbiota

The distribution of microorganisms in the gastrointestinal tract depends mainly on the pH gradient and the abundance of oxygen, and the changes in pH due to long-term PPI use are limited to the duodenum and proximal small intestine (Michalek et al., 2011), this part of the gastrointestinal tract is more closely related to the hepatobiliary and pancreas, bacteria may enter the body circulation through portal vein transfer and activate pro-inflammatory pathways organs (Gagnaire et al., 2017), which may induce solid tumor growth if these pathways are activated over time (Mantovani et al., 2008). When the inflammatory pathway is activated, it may lead to the abnormal metabolism of bile acids, thus inducing cholestatic liver cancer (Singh et al., 2018). Bacteria can disrupt the normal DNA repair by producing toxins and alter the bile acid metabolism process by enzymes on the surface of bacteria, thus leading to local inflammation and vascular proliferation in the tissues and increasing the possibility of biliary tract stone formation, which are potential biliary tumor carcinogenic mechanisms (Ward et al., 1994; Lara-Tejero and Galán, 2000; Haghjoo and Galán, 2004).

Long-term PPI reduces gastrointestinal microbial diversity by blocking gastric acid secretion and affecting gut microbiota diversity (Jackson et al., 2016; Zhernakova et al., 2016), it can increase the growth of potentially pathogenic bacteria such as Clostridium difficult, Enterococcus, Streptococcus, Staphylococcus, and E. coli (Jacobs et al., 2013; Su et al., 2018), as well as the disruption of the intestinal barrier and the alteration of intestinal permeability, these changes in the normal structure and microbiota can lead to excessive accumulation of lipopolysaccharides (LPS) and increased levels of deoxycholic acid in the tumor microenvironment, and hyper-deoxycholic acidemia can induce the development of HCC by damaging DNA (Bindels et al., 2012), LPS promotes HCC pathogenesis and metastasis and affects prognosis by upregulating toll-like receptor (TLR4) expression, thereby increasing cell proliferation, inhibiting apoptosis and producing a specific systemic inflammatory response. Activation of TLR2 by lipid wall phosphate in bacteria and ursodeoxycholic acid leads to upregulation of the senescence-associated secretory phenotype (SASP) and cyclooxygenase 2 (COX-2), which mediates prostaglandin 2 inhibition of antitumor immunity via EP4 receptors, thereby inducing HCC progression (Yoshimoto et al., 2013).

As an important secretory organ of the body, the pancreas requires the assistance of intestinal microorganisms for the application of its digestive enzymes. The antimicrobial activity of pancreatic fluid protects the pancreas from retrograde infection and contributes to the diversity of the gut microbiota. However, intestinal microorganisms can reach the pancreas via the circulatory system or the biliary/pancreatic duct, especially in the case of abnormal gut microbiota (Fritz et al., 2010). The current study suggests that the abnormal distribution of *Enterococcus faecalis* and *Escherichia coli* may be associated with the progression of pancreatic tumors associated with pancreatitis (Maekawa et al., 2018). Microorganisms promote tumor development, invasion and migration by activating the inflammatory response, increasing pro-inflammatory cell recruitment and cytokine secretion, increasing exposure to oxidative stress, altering energy dynamics, and damaging DNA, ultimately leading to molecular alterations and tumor transformation. In addition, chronic inflammation caused by non-pathogenic bacteria can induce the production of angiogenic factors, which increase the oxygen as well as nutrient supply to tumor and directly accelerate cancer cell growth. Alterations in several molecular mechanisms: oncogene mutations, oncogene inactivation, loss of heterozygosity, and chromosomal and microsatellite instability are also involved in inflammation-mediated oncogenesis. Cells within the microenvironment control tumor growth through the production of autocrine, paracrine, and endocrine mediators (Morgillo et al., 2018). It is believed that abnormal gut microbiota is an important cause of weight-related tumors, weight abnormalities aggravate the homogeneity of the gut microbiota by increasing deoxycholic acid production, which can lead to DNA damage, and activate the K-RAS pathway to induce pancreatic cancer (Schulz et al., 2014). LPS on the cytosolic surface of bacteria are involved in the progression and invasion of pancreatic cancer through a cascade reaction generated by LPS-TLR, but no studies have shown this mechanism increases the risk of pancreatic cancer (Yu et al., 2010; Dapito et al., 2012; Mishra et al., 2016).

Bile acid concentrations in the digestive tract are significantly higher in GERD patients receiving long-term PPI therapy than in healthy individuals (Stamp, 2002), Bile acids can directly disrupt the plasma membrane and cause activation of the PKC and p38 MAPK pathways, which result in a cascade reaction that activates the downstream IL-6 and Janus kinase (JAK)—signal transducer and activator of transcription 3 (STAT3) pathways, then leading to HCC (Nag et al., 2013), and persistently high bile acid levels can stimulate the development of HCC (Kong et al., 2016).

Insufficient gastric acid leads to microbiota translocation and overgrowth in the digestive tract leading to dysbiosis, and an increased pH leads to bacterially catalyzed N- nitrosamine leading to nitrosamine reduction and rapid nitrosamine production in the lumen. Faster nitrosation triggers the production of potentially carcinogenic N-nitrosamines in the digest tract (Yeomans et al., 1995). The association between nitrosamines and various types of cancer has been extensively studied (Mirvish, 1995). The association between nitrosamines and various types of cancer has been extensively studied (Rustagi and Dasanu, 2012), which can increase the risk of pancreatic cancer by affecting β2-AR signaling and upregulating HIF-1α expression (Zhang et al., 2016), it also caused DNA damage and decreased repair capacity in the pancreatic duct epithelium in synergy with glucagon (Howatson and Carter, 1987; Risch, 2012), however, a meta-analysis of the association between nitrosamine exposure and pancreatic cancer development did not report a direct association (Zhou et al., 2012; Fritschi et al., 2015). In hepatocellular carcinoma, nitrosamine induces apoptosis in human normal liver cell lines through endogenous and exogenous pathways of caspases (García et al., 2009), nitrosamine has been shown to induce hepatocellular carcinoma in mouse models (Balaraman et al., 2021). Therefore, it is reasonable to assume that nitrites produced by gut microbiota disorders have varying degrees of induction in Hepato-biliary-pancreatic cancer.

Long-term abnormal hormonal stimulation, decreased diversity of gut microbiota, production of carcinogenic substances, chronic inflammation, and activation of tumor pathways may all be biologically linked to long-term PPI use, and therefore the amplification of these biological mechanisms and the synergy between them should be further investigated and patients on long-term PPIs and with high risk factors for hepato-biliary-pancreatic cancer should have regular medical check-ups.

However, there is still a proportion of studies showing synergistic effects of PPI on chemotherapeutic agents for tumors. For example, PPIs show dose-dependent antitumor effects on esophageal cancer cells and breast cancer cells and enhance the sensitivity of tumor cells to chemotherapeutic agents (Matsumura et al., 1410; Ihraiz et al., 2020). This may be due to the fact that PPI cause acidification of the tumor microenvironment, which induces apoptosis, inhibits tumor cell migration and enhances the chemo-sensitivity of tumor cells (Falcone et al., 2016). However, no studies have shown a coordinated effect of PPIs on the chemotherapy of hepato-biliary-pancreatic cancers, therefore a lot of basic research is still needed to investigate the association between them.

Potassium-competitive acid blockers (P-CABs) are theoretically superior to PPIs due to its pH stability in digestive tract and long half-life period (Rettura et al., 2021), and have been shown to be non-inferior to PPIs for acid-related disorders in many clinical trials (Komori et al., 2019; Sunwoo et al., 2020). The efficacy and safety of potassium-competitive acid blockers should therefore be the focus of more research in the future. But at present, the great benefits of PPI treatment for patients appear to outweigh the risks of cancer. This risk of cancer can be avoided by following up high-risk patients and stopping PPI in time.

## Limitations

Although the results of this study corroborate the conclusion that long-term PPI may increase the risk of hepato-biliary-pancreatic cancer. However, there are still some shortcomings: if some measurement uniformities were available, we could have performed a dose or response duration analysis to evaluate the linear relationship, which would have helped quantify the association more accurately. and the dose and duration inconsistencies mentioned above may have contributed to this heterogeneity. Third, the relationship between long-term PPI use and biliary system cancer may be biased, the bias may be caused by the number of cDDD in the studies. In addition, the meta-analysis included only studies published in English, with smaller studies with cumulative results often unpublished, leading to potential biases.

## Conclusion

In conclusion, the results of this study corroborate the argument that the risk of hepatobiliary and pancreatic cancer is higher among IPP users. Whether the risk of cancer development was analyzed for hepato-biliary-pancreatic cancer or for single cancers, there was a positive correlation between the risk of tumor development and low doses, the risk of tumor development was highest at normal doses. However, no further increase in tumor risk was found with higher cumulative defined daily dose. Hence, further studies are needed to clarify and validate the mechanism. However, health professionals should carefully consider the prescription of PPI for patients at high risk of hepato-biliary-pancreatic cancer and control the misuse of medications.

## Data Availability

The original contributions presented in the study are included in the article/Supplementary Material, further inquiries can be directed to the corresponding author.

## Supplementary material

SUPPLEMENTARY FIGURE S1 The result of Egger’s test and trim and filling method.

SUPPLEMENTARY FIGURE S2 The result of sensitivity analysis of included studies.

SUPPLEMENTARY APPENDIX S1 Preferred reporting items for systematic reviews and meta-analyses (PRISMA) guidelines and the Cochrane handbook.

SUPPLEMENTARY APPENDIX S2 Search strategy details.
