# Supplementary material for: Using proton pump inhibitors increases the risk of hepato-biliary-pancreatic cancer. A systematic review and meta-analysis
Source: Front Pharmacol. 2022 Sep 14;13:979215. doi: 10.3389/fphar.2022.979215 (PMC9515471; doi:10.3389/fphar.2022.979215)
Supplement: Supplementary file 1 [file Table1.DOCX]

| First author | Country | Study period | Study design | Study participants | Definition and cancer | Confounder adjusted in the multivariate analysis | NOS |
| --- | --- | --- | --- | --- | --- | --- | --- |
| Xiong et al 2020 (13) | China | 2002-2018 | Case-control | 606/2424 | gallbladder cancer (GBC) | Infectious disease (HBV, HCV), Fatty liver disease, Alcohol intake, smoking, diabetes mellitus, Hypertension, Obesity, Coronary artery disease, Aspirin use, Dyslipoproteinaemia | 6 |
| Xiong et al 2020 (14) | China | 2002-2018 | Case-control | 1468/2936 | Intrahepatic cholangiocarcinoma (ICC)  Extrahepatic cholangiocarcinoma (ECC) | Infectious disease (HBV, HCV), Fatty liver disease, Alcohol intake, Smoking, Diabetes mellitus, Dyslipoproteinaemia, Hypertension, Obesity, Coronary artery disease, Aspirin use | 6 |
| Peng et al 2018 (15) | China | 2006-2011 | Case-control | 2293/2293 | Cholangiocarcinoma (ICD-9-CM). | Gastric polyp, Gastritis, Cirrhosis, Diabetes, Chronic pancreatitis, Hepatitis B/C, Inflammatory bowel disease, Biliary tract disease, Stroke, CAD, COPD, Alcohol-related illness, Clonorchis, Opisthorchis, HP | 8 |
| Kamal et al 2021 (30) | Sweden | 2005-2012 | Cohort | 738881 | Gallbladder cancer (ICD-10)  Extrahepatic cancer  Intrahepatic cancer | Gastroesophageal reflux, Peptic ulcers, Gastroduodenitis, HP, Chronic pancreas disease, Chronic liver disease, Gallstone disease | 9 |
| Lai et al 2013 (16) | China | 2000-2010 | Case-control | 3087/12348 | Liver cancer (ICD-9 codes 155, 155.0, 155.2, A- code A095) | diabetes mellitus, cirrhosis, alcoholic liver damage, nonalcoholic fatty liver disease, hepatitis B infection, hepatitis C infection, and tobacco | 7 |
| Tran et al 2018 (17) | UK | 1999-2011  2006-2010 | Case-control  Cohort | 434/2103  47576 | Liver cancer (Read codes: B15, excluding B153)  Liver cancer (ICD-10; C22) | diabetes, coronary heart disease, myocardial infarction, heart failure, peripheral vascular disease, cerebrovascular disease, cerebrovascular accident, chronic obstructive pulmonary disease, mental illness, GERD, peptic ulcer disease and hepatitis, cirrhosis, alcoholic fatty liver, non-alcoholic fatty liver, biliary cirrhosis | 7/9 |
| Kao et al 2019 (31) | China | 2003-2013 | cohort | 114984 | HCC (ICD-9-CM codes 155.0, 155.2) | diabetes mellitus, stroke, Cirrhosis, Nonalcoholic liver disease, Alcoholic liver disease, Hypertension, Chronic kidney disease, Hyperlipidemia | 7 |
| Li et al 2017 (32) | USA | - | cohort | 5752/5754 | Liver cancer (ICD-9-CM) | age, sex, race, smoking history, alcohol abuse history, body mass index, diabetes, baseline FIB-4 score, gastroesophageal reflux disease, HCV genotype, past completed anti-HCV treatment | 8 |
| Shao et al 2018 (18) | China | 2000-2013 | Case-control | 29473/29450 | Liver cancer (ICD-9-CM; 155.0) | hypertension, diabetes, COPD, acute coronary syndrome, cerebrovascular accident, peptic ulcer disease, GERD, cirrhosis, hyperlipidemia, HP eradication therapy, H2-receptor antagonists, aspirin, NSAIDs. | 7 |
| Kim et al 2022 (33) | Korea | 2003-2006 | Cohort | 406057 | HCC (ICD-10, c220) | age, sex, household income, charlson comorbidity index, systolic blood pressure, diastolic blood pressure, BM, fasting serum glucose, cigarette smoking, alcohol consumption, physical activity | 7 |
| Lee et al 2020 (19) | USA | 1996-2016 | Case-control | 2329/19987  567/4820 | Liver cancer  Pancreatic cancer | chronic hepatitis B/C, hemochromatosis, alpha-1 antitrypsin deficiency, cirrhosis, diabetes mellitus, glycogen storage disease, gastrointestinal bleeding, fatty liver disease, autoimmune hepatitis, cystic fibrosis, chronic pancreatitis, diabetes mellitus, and pancreatic cysts. | 7 |
| Brusselaers et al 2020 (34) | Sweden | 2005-2012 | Cohort | 796492 | Pancreatic cancer (ICD-10) | age, indications for gastric acid suppressive therapy, diabetes | 9 |
| Peng et al 2018 (20) | China | 2006-2011 | Case-control | 1087/1087 | Pancreatic cancer (ICD-9-CM) | Age, chronic pancreatitis, biliary tract disease | 6 |
| Hick et al 2018 (21) | Denmark | 2000-2015 | Case-control | 6921/34605 | Pancreatic cancer (ICD-10) | Diabetes, alcohol-related disease, COPD, chronic pancreatitis, gallstones, peptic ulcer, Helicobacter pylori infection, hepatitis B and C infection, use of low-dose aspirin, NSAIDs, statins, HRT, CCI, highest achieved education | 7 |
| Hwang et al 2018 (35) | Korea | 2002-2013 | Cohort | 453655 | Pancreatic cancer (ICD-10) | Age, BMI, smoking, alcohol, drinking, physical activity, diabetes, chronic pancreatitis, CCI, SE | 9 |
| Kearn et al 2017 (22) | UK | 1995-2013 | Case-control | 4113/16072 | Pancreatic cancer | Diabetes, smoking, alcohol, obesity | 7/9 |
| Boursi et al 2017 (36) | UK | 1995-2013 | Cohort | 19146 | Pancreatic cancer | NA | 5 |
| Lai et al 2014 (23) | China | 2000-2010 | Case-control | 977/3908 | Pancreatic cancer (ICD-9) | Acute pancreatitis, chronic pancreatitis, diabetes, obesity, H2RA, statin, non-statin lipid-lowering, both ASA and COX2i | 7 |
| Bosetti et al 2013 (24) | USA/Canada/Australia | - | Case-control | 56/51 | Pancreatic cancer | NA | 6 |
| Bradley et al 2012 (25) | UK | 1995-2006 | Case-control | 1141/7954 | Pancreatic cancer | Smoking, BMI, alcohol, history of chronic pancreatitis, use of other drugs (NSAIDs, steroids, HRT), diabetes, prior cancer | 7 |
| Lassalle et al 2022 (26) | French | 2014-2018 | Case-control | 23321/75937 | Pancreatic cancer (ICD-10; ref. 25) | diabetes mellitus, tobacco-related diseases (including COPD diagnosis) or drug use,  morbid obesity, alcohol-related diseases or drug use, acute pancreatitis, chronic pancreatitis, pancreatic cyst, gallstones, hepatitis B or C, peptic ulcer, Helicobacter pylori eradication, myocardial infarction, congestive heart failure, peripheral vascular disease, cerebrovascular disease, dementia,  chronic obstructive pulmonary disease, connective tissue disease, mild liver disease, hemiplegia, moderate to severe liver disease, chronic kidney disease, HIV/AIDS, antihypertensive drug use, NSAID use, statin use | 7 |
| Valente et al 2017 (27) | Italy, Norway, Sweden, Slovenia, UK,  Germany | 2013-2015 | Case-control | 201/603 | malignant neoplasm of the pancreas (ICD-Oncology C25.0-C25.9) | Acute pancreatitis, Chronic pancreatitis, Peptic ulcer, Cholecystectomy, Gastrectomy, Gallstone disease, Asthma, Eczema, Hay fever, Any allergy, Use of aspirin | 8 |
| Chien et al 2016 (28) | China | 2000-2010 | Case-control | 7681/76762 | extrahepatic cholangiocarcinoma (ICD-O-3: C24.0), ampullary (ICD-O-3: C24.1), duodenum (ICD-O-3: C17.0), jejunum (ICD-O-3: C17.1), pancreatic (ICD-O-3: C25.0). | Choledochal cysts, cholangitis, cholelithiasis, cirrhosis, alcoholic liver disease, NAFLD, HBV, HCV, diabetes, chronic pancreatitis, inflammatory bowel disease, PUD, GERD, cardiovascular diseases | 7 |
| Lin et al 2020 (37) | China | 2001-2005 | Cohort | 164167 | Hepatocellular carcinoma (ICD-9) | Age, gender, viral hepatitis, chronic liver disease and cirrhosis, alcohol abuse, obesity, diabetes mellitus, schistosomiasis parasitic infection, tobacco use disorder, statin use, thiazolidinedione use, and metformin use | 9 |
| Risch et al,2015 (29) | USA | 2005-2009 | Case-control | 895 | Pancreatic cancer | cigarette smoking and other tobacco use, alcoholic  beverage consumption, usage of medications for relief of  heartburn or acid reflux, usual adult height and weight, family  history of pancreatic cancer, Jewish ancestry, and diagnoses  of pancreatitis and diabetes mellitus | 6 |

TableS1: Baseline information of included studies
