# Supplementary figures and images for: Using proton pump inhibitors increases the risk of hepato-biliary-pancreatic cancer. A systematic review and meta-analysis
Source: Front Pharmacol. 2022 Sep 14;13:979215. doi: 10.3389/fphar.2022.979215 (PMC9515471; doi:10.3389/fphar.2022.979215)

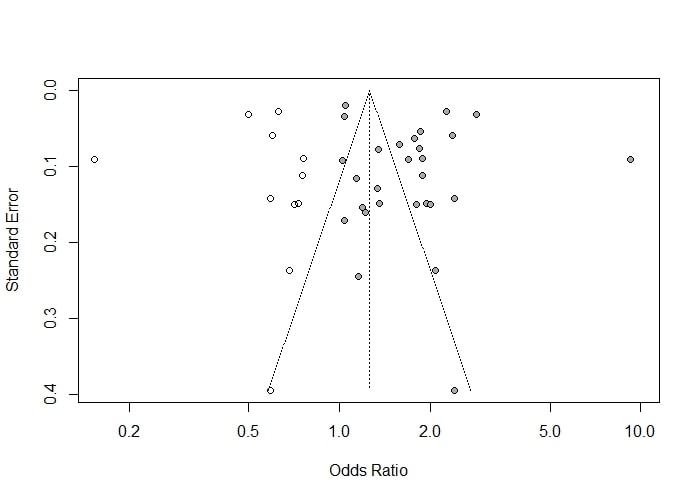

Supplement: Supplementary file 2 [file Image1.JPEG]

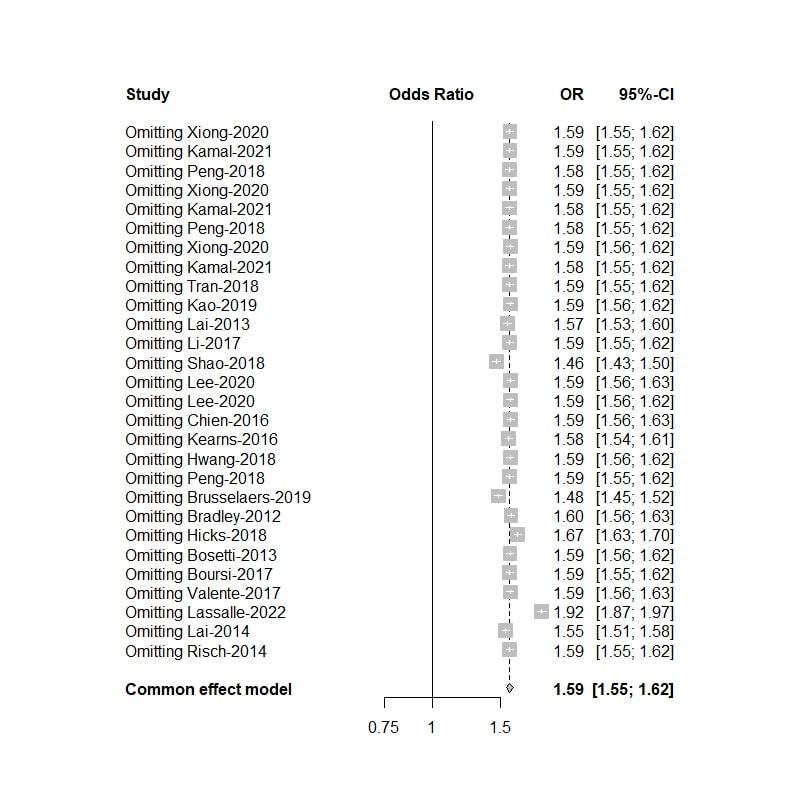

Supplement: Supplementary file 3 [file Image2.JPEG]
