# Supplementary material for: Using proton pump inhibitors increases the risk of hepato-biliary-pancreatic cancer. A systematic review and meta-analysis
Source: Front Pharmacol. 2022 Sep 14;13:979215. doi: 10.3389/fphar.2022.979215 (PMC9515471; doi:10.3389/fphar.2022.979215)
Supplement: Supplementary file 4 [file Table2.DOCX]

| Study | Selection | | | | Comparability | | Exposure | | |  |
| --- | --- | --- | --- | --- | --- | --- | --- | --- | --- | --- |
| (Author, year) | Adequacy | Representiveness | Selection | Definition | Main factors | Additional factors | Ascertainment | Method | Non-response | Quality |
| Peng et al 2018 (15) | - | * | * | - | - | * | * | * | - | 6 |
| Xiong et al 2020 (13) | - | * | - | * | * | * | * | * | - | 6 |
| Xiong et al 2020 (14) | - | * | - | * | * | * | * | * | - | 6 |
| Lai et al 2013 (16) | - | * | * | * | * | * | * | * | - | 7 |
| Bradley et al 2012 (25) | - | * | * | * | * | * | * | * | - | 7 |
| Lai et al 2014 (23) | - | * | * | * | * | * | * | * | - | 7 |
| Kearns et al 2017 (22) | - | * | * | * | * | * | * | * | - | 7 |
| Tran(PCCIU) et al 2018 (17) | - | * | * | * | * | * | * | * | - | 7 |
| Lee et al 2020 (19) | - | * | * | * | * | * | * | * | - | 7 |
| Shao et al 2018 (18) | - | * | * | * | * | * | * | * | - | 7 |
| Chien et al 2015 (28) | - | * | * | * | * | * | * | * | - | 7 |
| Peng et al 2018 (20) | - | * | * | * | - | * | * | * | - | 6 |
| Hick et al 2018 (21) | - | * | * | * | * | * | * | * | - | 7 |
| Bosetti et al 2013 (24) | - | - | * | * | * | * | * | * | - | 6 |
| Valente et al 2017 (27) | * | * | * | * | * | * | * | * | - | 8 |
| Lassalle et al 2022 (26) | - | * | * | * | * | * | * | * | - | 7 |
| Risch et al 2015 (29) | - | * | * | - | - | * | * | * | - | 6 |

A: Case-control study

| study | Selection |  |  | comparability |  | Outcome |  |  |  |
| --- | --- | --- | --- | --- | --- | --- | --- | --- | --- |
| Author, year | representiveness | selection | ascertainment | Main factor | Additional factor | assessment | Follow-up | adequacy | quality |
| Kamal et al 2021 (30) | * | * | * | * | * | * | * | * | 9 |
| Kao et al 2018 (31) | - | * | * | * | * | * | - | * | 7 |
| Hwang et al 2018 (36) | * | * | * | * | * | * | * | * | 9 |
| Tran(UK biobank) et al 2018 (17) | * | * | * | * | * | * | * | * | 9 |
| Brusselaers et al 2019 (34) | * | * | * | * | - | * | * | * | 9 |
| Li et al 2017 (32) | - | * | * | * | * | * | * | * | 8 |
| Boursi et al 2017 (35) | - | - | * | - | * | * | * | * | 5 |
| Kim et al 2022 (33) | * | * | * | * | - | * | * | * | 7 |
| Lin et al 2020 (37) | * | * | * | * | * | - | * | * | 9 |

B: Cohort study

Table S1: Quality assessment and New-castle Ottawa scale scores of the included studies (A: Case-control study; B: Cohort study)
