# Supplementary material for: Using proton pump inhibitors increases the risk of hepato-biliary-pancreatic cancer. A systematic review and meta-analysis
Source: Front Pharmacol. 2022 Sep 14;13:979215. doi: 10.3389/fphar.2022.979215 (PMC9515471; doi:10.3389/fphar.2022.979215)
Supplement: Supplementary file 5 [file Table5.DOCX]

Pubmed: ((((Proton Pump Inhibitor) AND ((Biliary Tract Neoplasm) OR (Neoplasm, Biliary Tract) OR (Biliary Tract Cancer) OR (Cholangiocarcinomas) OR (Cholangiocellular Carcinoma) OR (Extrahepatic Cholangiocarcinoma) OR (Intrahepatic Cholangiocarcinoma) OR (Gallbladder Neoplasm) OR (Gallbladder Cancer) OR (Gall Bladder Cancer) OR (BTC) OR (CC) OR (GBC))) NOT (review [Publication Type])) NOT (comment [Publication Type])) NOT (letter [Publication Type])

((((Carcinomas, Hepatocellular) (Hepatocellular Carcinomas) (Liver Cell Carcinoma, Adult) (Liver Cancer, Adult) (Adult Liver Cancer) (Adult Liver Cancers) (Carcinomas, Liver Cell) (Cell Carcinoma, Liver) (Liver Cell Carcinomas) (Hepatocellular Carcinoma) (Hepatomas) (HCC)) AND ((Proton Pump Inhibitor))) NOT (comment[Publication Type])) NOT (letter[Publication Type])) NOT (review[Publication Type])

Embase: 'proton pump inhibitor'/exp AND ('bile duct carcinoma'/exp OR 'biliary tract cancer'/exp OR 'cholangiocarcinoma cell line'/exp)

'Proton pump inhibitor'/exp AND ('hepatocellular carcinoma cell line'/exp OR 'liver cancer'/exp)

Cochrane library:

ID Search Hits

#1 MeSH descriptor: [Biliary Tract Neoplasms] explode all trees 496

#2 MeSH descriptor: [Gallbladder Neoplasms] explode all trees 91

#3 MeSH descriptor: [Cholangiocarcinoma] explode all trees 251

#4 MeSH descriptor: [Proton Pump Inhibitors] explode all trees 1551

#5 (Biliary Tract Neoplasm) OR (Neoplasm, Biliary Tract) OR (Biliary Tract Cancer) OR (Cholangiocarcinomas) OR (Cholangiocellular Carcinoma) OR (Extrahepatic Cholangiocarcinoma) OR (Intrahepatic Cholangiocarcinoma) OR (Gallbladder Neoplasm) OR (Gallbladder Cancer) OR (Gall Bladder Cancer) OR (BTC) OR (CC) OR (GBC):ti,ab,kw 20863

#6 #1 OR #2 OR #3 OR #5 21140

#7 #4 AND #6 24

(Pancreatic Neoplasm) OR (Pancreas Neoplasm) OR (Cancer of Pancreas) OR (Pancreas Cancer) OR (Pancreatic Cancer) OR (Pancreatic endocrine tumor) OR (Periampullary carcinoma) OR (VPC)

(Stomach Neoplasm) OR (Gastric Neoplasm) OR (Cancer of Stomach) OR (Stomach Cancers) OR (Gastric Cancer) OR (Cancer of the Stomach) OR (Gastric Cancer, Familial Diffuse)

(Duodenal Neoplasm) OR (Duodenal Cancer) OR (Cancer of the Duodenum) OR (Duodenum Cancer)

(Colorectal Neoplasm) OR (Colorectal Tumor) OR (Colorectal Cancer) OR (Colorectal Carcinoma)
