# Supplementary material for: Using proton pump inhibitors increases the risk of hepato-biliary-pancreatic cancer. A systematic review and meta-analysis
Source: Front Pharmacol. 2022 Sep 14;13:979215. doi: 10.3389/fphar.2022.979215 (PMC9515471; doi:10.3389/fphar.2022.979215)
Supplement: Supplementary file 6 [file Table3.DOCX]

| **Stratified by** | n | OR(95%CI) | P value |
| --- | --- | --- | --- |
| **Study design** |  |  |  |
| Case-control |  | 1.73（1.39，2.15） |  |
| cohort |  | 1.63(1.37,1.95) |  |
| **Region** |  |  |  |
| Asia |  | 1.67（1.43，1.96） |  |
| western |  | 1.53(1.31,1.78) |  |
| **NOS score** |  |  |  |
| ＜7 |  | 1.79（1.59，2.02） |  |
| ≥7 | 24 | 1.66（1.36，2.04） |  |

|  | n | OR （95%CI） | I2 | P |
| --- | --- | --- | --- | --- |
| **Liver cancer** |  |  |  |  |
| Asia |  | 1.75（1.39-2.21） | 94% | ＜0.01 |
| western |  | 1.66(1.30,2.13) | 77% | ＜0.01 |
| **Hepatocellular carcinoma** |  |  |  |  |
| Asia |  | 1.66(1.12-2.45) | 96% | ＜0.01 |
| western |  | 1.57(1.05-2.35) | 85% | ＜0.01 |
| **Biliary tract cancer** |  |  |  |  |
| Asia |  | 2.19(1.8-2.67) | 52% | 0.08 |
| western |  | 1.73(1.57-1.89) | 24% | 0.27 |
| **Pancreatic cancer** |  |  |  |  |
| Asia |  | 1.67（1.29-1.77） | 77% | ＜0.01 |
| western |  | 1.45(1.17-1.78) | 99% | ＜0.01 |

| **pooled estimates.** |  |  |  |  |
| --- | --- | --- | --- | --- |
| Studies report odds ratios |  | 1.76（1.47-2.10） | 98% | ＜0.01 |
| Studies report hazard ratios |  | 1.41（1.05-1.89） | 77% | ＜0.01 |

Table S2: Subgroup analysis and meta-regression in different subgroups
